# Transitions of care between pediatric onco-hematology and intensive care units: An exploratory study of healthcare professionals’ perceptions and practices in medication management

**DOI:** 10.1016/j.rcsop.2026.100789

**Published:** 2026-04-24

**Authors:** C. Baudy, O. Kouassi, M. Philippe, C. Gervaise, S. Remy, E. Javouhey, B. Dumont, C. Halfon-Domenech, V. Bréant, D. Hoegy

**Affiliations:** a Pharmacy, Groupement Hospitalier Est, Hospics Civils of Lyon, Lyon, France; b Hematology and Oncology Pediatric Institut, Lyon, France; c Paediatric Intensive Care Unit, Mother and Children University Hospital, Hospics Civils of Lyon, Bron, France; d UR 4129 P2S Parcours Santé Systémique, Healthcare Systemic Pathway, Univ Claude Bernard, Univ Lyon 1, Lyon, France; e Medication Sciences Department – Clinical pharmacy, Pharmaceutical and Biological sciences Institut, University Claude Bernard Lyon 1, Lyon, France

**Keywords:** Transition of care, Pediatric hematology-oncology, Pediatric intensive care, Drug management

## Abstract

**Background:**

As in adult care, pediatric patients experience transitions in their care trajectory, such as transfers to intensive care units (ICUs) during episodes of clinical deterioration. In pediatric onco-hematology, the administration of Chimeric Antigen Receptor (CAR) T-cell therapy often necessitates several days of ICU admission for post-infusion monitoring. As a result, systematic collaboration between pediatric onco-hematology and intensive care teams is becoming standard for these advanced therapies.

**Objective:**

This study aimed to explore healthcare professionals' perceptions, experiences, and practices related to medication management during transitions of care between pediatric onco-hematology and ICU settings.

**Methods:**

Semi-structured interviews were conducted with healthcare professionals from both specialties. Transcripts were analyzed using a manual inductive approach.

**Results:**

Twenty-four healthcare professionals participated (14 ICU, 10 onco-hematology). Themes emerged: (1) the clinical complexity of onco-hematology patients, (2) technical and logistical barriers (e.g., software incompatibility, clinical transfers, medication transfer), (3) the specific involvement of parents in pediatric care. Emergency ICU admissions were identified as the highest-risk transfer for medication-related adverse events. Improvements included three essential areas: information diffusion especially improving written one and verbal nursing transmissions, implementing trainings and practices standardization by instance for intravenous medications. Clinical pharmacists can help in those improvement areas and so need to be implemented in pediatric ICU and work on those transitions to reduce medication errors.

**Conclusion:**

These findings underscore the need to optimize medication safety during care transitions. Strategic efforts such as interdisciplinary collaboration, standardized protocols, and pharmacist involvement can help reduce risks and improve outcomes.

## 1 Introduction

In pediatrics, medication management poses a heightened risk of error, particularly during transitions of care. Similar to adult settings, pediatric care pathways often involve transfers between departments, such as from a general ward to an intensive care unit (ICU), typically prompted by a deterioration in the child's clinical condition. These transitions are especially vulnerable to medication-related errors due to the handoff between different healthcare teams and, in some cases, the lack of integrated electronic health records.^1^, ^2^

In pediatric onco-hematology, the emergence of Advanced Therapy Medicinal Products (ATMPs)—notably Chimeric Antigen Receptor (CAR) T-cell therapy—has introduced new challenges. These treatments often require ICU admission for post-infusion monitoring, thereby systematizing a transfer that was previously reserved for patients with acute deterioration.

This study specifically examines how medications are managed during these transitions. As a first step toward identifying areas for improvement, we explored the perceptions, experiences, and practices of healthcare professionals (HCPs) involved in these transitions between pediatric onco-hematology and ICU services.

## 2 Methods

This study was conducted at two geographically distinct hospital sites in France: one providing pediatric onco-hematology care and the other housing a pediatric intensive care unit (ICU). Semi-structured interviews were conducted by two researchers trained in qualitative research methodology. In order to study each center as an ecosystem over the same period of time, one researcher interviewed participants from the pediatric onco-hematology unit, while the other interviewed participants from the pediatric ICU. The onco-hematology interviews were conducted by a physician pursuing a master's degree, and the ICU interviews by a hospital pharmacy resident. Neither researcher had prior professional experience working in pediatric onco-hematology or ICU settings.

### 2.1 Participants

Eligible participants were healthcare professionals (HCPs)—including physicians, pharmacists, nurses, and health executives—from either site who had experience managing a patient during a transition of care between the pediatric onco-hematology unit and the ICU, in either direction.

### 2.2 Data collection

Before recruitment began, two interview guides were developed by a researcher with expertise in qualitative methods (see Table 1, Table 2). Interviewers did not know the participants before interviews and presented themselves as researchers to the participants. Each guide was pilot-tested and revised accordingly. The interviews explored four main themes: professional experience, communication practices, perceived barriers and facilitators, and areas for improvement.

**Table 1:** Interview guide for HCPs in pediatric ICU.

Key themes	Questions
**Experience**	When a patient arrives in the Intensive Care Unit (ICU) who was being treated in the onco-hematology department, how did the patient's medication management go? (including all therapeutics)
	*Rephrasing: When you received a patient from the onco-hematology department, how was his or her medication managed on arrival? At discharge?*
**Communication**	In your opinion, what do you need in terms of information to best manage the patient admitted to the ICU?
	In your opinion, what do HCPs need in terms of information to provide the best possible care for patients at ICU discharge?
	*Prompt: Joint care plan? Care history? Medication history? Medication analysis / reconciliation? History and allergies? Objectives and preferences? Contact details?*
**Barriers and facilitators**	What barriers do you see to effective medication management during this transition?
	What facilitators do you see for the most effective medication management during this transition?
	*Prompt: in the following direction onco-hematology care – ICU, then ICU – onco-hematology care*
**Areas for improvement**	What areas for improvement would you envision to ensure safe medication management during this transition?
	*Prompt: in the following direction onco-hematology care – ICU, then ICU – onco-hematology care*
**Synthesis**	Would you like to add anything else?
	*Rephrasing of what has been said (brief summary)*

**Table 2:** Interview guide for HCPs in the onco-hematology unit.

Key themes	Questions
Experience	How do you manage patients referred to you by the ICU?
	When you transferred a patient to intensive care, how did the care management go?
	*Rephrasing: When you received a patient from the ICU, how did the continuity of care or medication transition go? When you transferred a patient to the ICU, how was the transfer of care managed?*
Communication	In your opinion, what information do you need to better manage the patient coming from the ICU (when admitted to the pediatric onco-hematology unit)? In your opinion, what do ICU physicians need in terms of information to better manage patients coming from the onco-hematology unit (at ICU admission)? *Prompt: Joint care plan? Care history? Medication history? Medication analysis / reconciliation? History and allergies? Objectives and preferences? Contact details?*
Barriers and facilitators	What are the barriers to efficient (or effective) medication management during the transition from onco-hematology to intensive care, and then from ICU to onco-hematology?
	What are the facilitators to medication management during these transitions?
Areas for improvement	In your opinion, what needs to be improved to ensure safe medication management during these transitions? in the following direction: from onco-hematology to ICU? then from intensive care to onco-hematology?
Synthesis	Would you like to add anything else?
	Is there an element you deem important that we did not discuss?
	*Rephrasing of what has been said (brief summary)*

Lists of eligible participants from both the onco-hematology and ICU teams were compiled by the expert researcher. Invitations to participate were then sent via email.

Interviews were conducted individually in private settings, either in person during working hours or via video call outside working hours. All interviews were audio-recorded. Recruitment continued until the study lead determined that data saturation had been reached for each interviewer.^3^ Saturation was defined by the recurrence of at least one idea across four distinct categories in three consecutive interviews, as well as by the depth and richness of the data collected. Meaning saturation, for example, was considered achieved when new participants discussed topics already raised by previous participants (such as paper-based communication or patient transport) without introducing new insights (such as detailing or explaining).

### 2.3 Data analysis

Interviews from each group were analyzed separately using a manual inductive approach.^4^ Each researcher analyzed the transcripts of their own interviews in three stages: (1) reading the transcripts in full, (2) organizing data into thematic categories, and (3) selecting the most relevant verbatim excerpts to structure and illustrate each category. Themes were organized based on their recurrence across interviews. The two researchers then compared their analyses and resolved any discrepancies through consensus. By jointly reviewing their respective analysis reports, they assessed similarities and differences and agreed on a final, harmonized analysis report using consistent terminology.

No formal comparative analysis was conducted between the two groups. An expert researcher reviewed the results, refining the categories and subcategories across both groups. A final review of the findings allowed for further reorganization of categories and subcategories using the interpretative framework of Normalization Process Theory.^5^ The finalized categories and subcategories were presented in tabular format and illustrated with representative participant quotations.

### 2.4 Ethics

Prior to data collection, the study was registered and conformed to the requirements of the French National Commission on Informatics and Liberties (CNIL), under registration number 22–102. Each participant received an information sheet explaining the study, and verbal consent was requested before the interviews were recorded. All data were anonymized and used solely for research purposes. Data were securely stored for five years in compliance with data protection regulations, within the hospital's information system and protected by individual password access restricted to the study's authorized personnel only.

## 3 Results

### 3.1 Participants

Twenty-four healthcare professionals participated between January and May 2023: 14 from the ICU and 10 from the onco-hematology unit. Among them, 10 were physicians, 8 were nurses, 1 was a chief nurse executive, and 4 were pharmacists. Their sociodemographic characteristics are presented in Table 3. Twenty-one interviews were conducted face-to-face and three via video call. The average length of experience in their specialty (excluding pharmacists) was 11 years, ranging from 2 to 25 years.

**Table 3:** Socio-demographic HCP characteristics.

HC	Profession	Gender	Intensive Care Unit	Onco-hematology	Years of experience
HC1	Physician	F	X		5
HC2	Nurse	F	X		18
HC3	Chief nurse Executive	F	X		7
HC4	Nurse	F	X		2.5
HC5	Nurse	M	X		2
HC6	Nurse	F	X		12
HC7	Physician	F	X		15
HC8	Physician	F	X		19
HC9	Physician	F	X		4.5
HC10	Pharmacist	F	X		Non applicable
HC11	Pharmacist	F	X		Non applicable
HC12	Physician	F	X		15
HC13	Pharmacist	F	X		NA
HC14	Nurse	F	X		2
HC15	Physician	F		X	1
HC16	Physician	M		X	2
HC17	Nurse	F		X	8
HC18	Physician	F		X	13
HC19	Pharmacist	M		X	Non applicable
HC20	Nurse	F		X	12
HC21	Physician	M		X	20
HC22	Nurse	F		X	13
HC23	Physician	M		X	6
HC24	Physician	M		X	6

The interviews lasted an average of 29 min, with durations ranging from 14 to 52 min.

### 3.2 Perceptions, experiences and practices

The perceptions, experiences, and practices of HCPs in the pediatric onco-hematology unit and pediatric ICU are summarized in Table 4.

**Table 4:** Identified perceptions, experiences and practices.

Specificities of patients from onco-hematology	
• Fear of iatrogenic medication: specific treatments, multiple treatments, rare care management, recent changes	
• Protective isolation	
• Different management between emergency care and scheduled care	
**Facilitators**	**Barriers**
• Close collaboration	• Difference in patient records and prescription software
• Documents and procedures to help patients make the transition to ICU	
	• Insufficient and time-consuming handover to the ICU
• Helpful medical experiences	• Suboptimal transportation
**Pharmaceutical practices and medication availability**	
• Different practices: drug preparation and administration, different therapeutic booklets	
• Different medication availability	
• Transferring medications between the two care units	
**The special role played by parents**	
• Differences in care management between onco-hematology and ICU	
• Strong involvement in patient care	

#### 3.2.1 Specificities of onco-hematology patients

Three ICU professionals expressed concern about the **potential for drug-related adverse events**, attributing this to their **limited experience** managing onco-hematology patients. Other elements were also brought up, potential factors explaining their fears. Fifteen professionals noted the complexity and **quantity of medications** involved in onco-hematology care.

*“What differs (...) is the number of treatments. Unfortunately, children (in onco-hematology) often have a huge number of treatments. “* HC6.

Four professionals also highlighted the use of **uncommon or specialized drugs**, which require experience to manage properly (HC1).

*“ Someone who's never seen it before may be surprised because they don't know the doses—either thinking it's too much, like with high-dose methotrexate, or too little, as with intrathecal injections.”* HC13.

One nurse highlighted the challenges of providing care to patients requiring **protective or sterile isolation**, particularly the additional time and effort associated with donning protective equipment before entering the room*“As soon as you enter a protective or sterile environment, you have to get dressed. (…) you don't usually go there for no reason”* HC2.

**Differences between emergency and scheduled care were highlighted.**. All the participants spontaneously shared their experiences with emergency transfers.

*“It's true that we also transfer in a hurry, so sometimes there can be missing information.”* HC17.

By contrast, scheduled **CAR T-cell therapy transfers were perceived as smoother** and more organized: “simpler” (HC1), “no particular organizational concerns” (HC3).

#### 3.2.2 Differences in drug dispensation, preparation and administration

Three ICU professionals reported challenges related to medication availability. These included the preparation of chemotherapy agents—rarely used in the ICU—and delays in obtaining certain medications, particularly overnight, which could result in delayed administration*.*

*“ We can't afford to stock all the onco-hematology drugs. There are just too many that we don't use daily.”* HC10.

Two pharmacists reported challenges related to the transfer of medications between departments. While such transfers may help nurses avoid delays in administration, they can complicate the pharmaceutical supply chain, particularly for high-cost medications. *“ ... Sometimes antifungals end up in emergency care settings, even though they don't belong there”* HC11.

Four ICU professionals reported **differences in how drugs are prepared and administered** between the two units. They pointed out differences between the onco-hematology unit, where drugs are often administered together, and intensive care, where more precautions are taken and drugs are more often administered separately.: *“ it seems […] that everything's gone at once, that it's all mixed up with the antibiotics, whereas for us, we try to get a central venous catheter for such and such... and work out the flow duration, the parenteral nutrition, recalculate the flow rates. “* HC6.

Three ICU nurses described challenges related to managing intravenous drug compatibility. A pharmacist noted that discrepancies in therapeutic preparation formularies could lead to confusion and require clarification between teams.

#### 3.2.3 Barriers

Healthcare professionals across all roles frequently mentioned a variety of barriers that complicate patient management during transitions between the pediatric onco-hematology unit and the ICU.

Two ICU physicians cited **differences in prescribing software** as a major obstacle, noting that they often have to use unfamiliar systems during clinical transfers. *“We're not proficient with the other system.”* – HC12.

Five professionals, including physicians and pharmacists, also reported limited access to digital patient records, particularly onco-hematology laboratory results, which could lead to information gaps during care transitions*“ The problem is that the two hospitals have different computer systems. So, transfers require paper-based exchanges since we can't access each other's records.”* HC21.

Six HCPs from various roles highlighted difficulties with the **paper-based transfer** process. These documents are often **difficult to interpret, time-consuming**, and, according to two participants, prone to error.

*“ If we can't complete the hospitalization summary clearly before transfer, we fax the remaining information within 10 minutes after the patient leaves.”* HC23.

Five ICU professionals and two from onco-hematology reported that information is often lost during transfer, particularly regarding the most recent medication administration times.

*“ It takes a huge amount of time to figure it all out. We often end up calling the onco-hematology team to clarify when antibiotics or other treatments were last given. “* HC1.

This problem also occurs when patients return from the ICU to onco-hematology. Three ICU nurses noted that the return transfer to the onco-hematology unit is not “intuitive” (HC14) or “well-adapted” (HC2). One nurse highlighted the **lack of documentation on drug administration** routes, which poses issues during sampling for residual levels:

*“It's rarely specified whether a sample was taken from a central line or elsewhere. And if we don't know, we can't draw from the catheter.”* – HC6.

Another reported barrier concerned patient transport. One ICU physician noted that patients were **not always transferred with emergency medical technicians (EMTs)** and that the child's clinical condition prior to ICU admission was often unknown. During transport, medications might not be administered and other aspects of care could be delayed or omitted. Onco-hematology professionals also expressed frustration with **delays related to reliance on the SAMU** (the French emergency medical service), particularly in emergency situations.

*“Sometimes it's fast, but being in a hospital doesn't give us priority. Transfers can be quick or take one to two hours (to go to the ICU).”* HC22.

#### 3.2.4 Facilitators

Seven ICU professionals and five onco-hematology professionals emphasized the strong collaboration between teams. They reported **effective communication** and the ongoing **involvement of the onco-hematology team in decision-making**, even after transfer.

*“ we're in regular contact (between ICU and onco-hematologist physicians) —even outside of emergencies—with a designated physician, and there are continuous efforts to improve things together… And the ICU physician is always reachable by phone in emergencies “* HC23.

The **daily visits of onco-hematology physicians to the ICU** were also seen as a key asset.

However, one pharmacist noted that having **too many points of contact** can actually complicate communication. In their view, the current system sometimes **lacks flexibility or space** for improvement in medication management.

*“It's a vicious circle—you call the onco-hematology pharmacist, then the physician, then the ICU physician”* HC11.

Four onco-hematology professionals noted that prior ICU experience among staff members (HC20) was beneficial during patient transfers.

*“Some residents or nurses have prior ICU experience or internships. These people are especially helpful during transfers because they take more initiative.”* HC20.

Additionally, two onco-hematology professionals suggested that joint interdisciplinary working groups—particularly those focused on life-threatening emergencies—could serve as a valuable platform for improving coordination and care transitions.*“We have working groups on emergency response... to train us in first aid, oxygen administration, using the emergency cart—skills we need in the onco-hematology department”* HC20.

Four onco-hematology professionals highlighted the **usefulness of a “liaison” sheet**, while three mentioned a **standardized resuscitation protocol** that is available to all medical staff.

*“ We have well-designed protocols [in the onco-hematology unit] that are currently being updated. They're clear, part of supportive care, and every intern and attending carries them.”* HC24.

#### 3.2.5 The specific role played by parents

Six healthcare professionals highlighted the unique role that parents play in the care of pediatric onco-hematology patients.

Four ICU professionals discussed how parents experience the transfer from onco-hematology to the ICU. They described a **perceived contrast** between the onco-hematology unit, seen as a “live-in unit”, and the ICU, seen more as a “treatment unit” (HC14). The transition often comes with multiple changes—new physical environment, different healthcare teams, altered routines, and a worsening clinical situation—which can be **distressing for parents**.

*“Multiple prescription errors and changes in routine create additional stress for parents.”* HC1.

Three ICU professionals emphasized the **importance of reassuring parents** during this time. Building trust through collaboration and effective communication between teams can help reduce parental anxiety.

*“The more they feel we've communicated and know their child well, the more reassured they are.”* HC1.

Two ICU nurses highlighted how involved and knowledgeable parents are, especially regarding their **child's medication** and care routines. This active participation can positively influence both care delivery and the **child's acceptance** of treatment. *“The parents from the onco-hematology unit are very observant. They're a huge help with many things and know every medication their child takes.”* HC6.

### 3.3 Areas for improvement

**Fig. 1:**
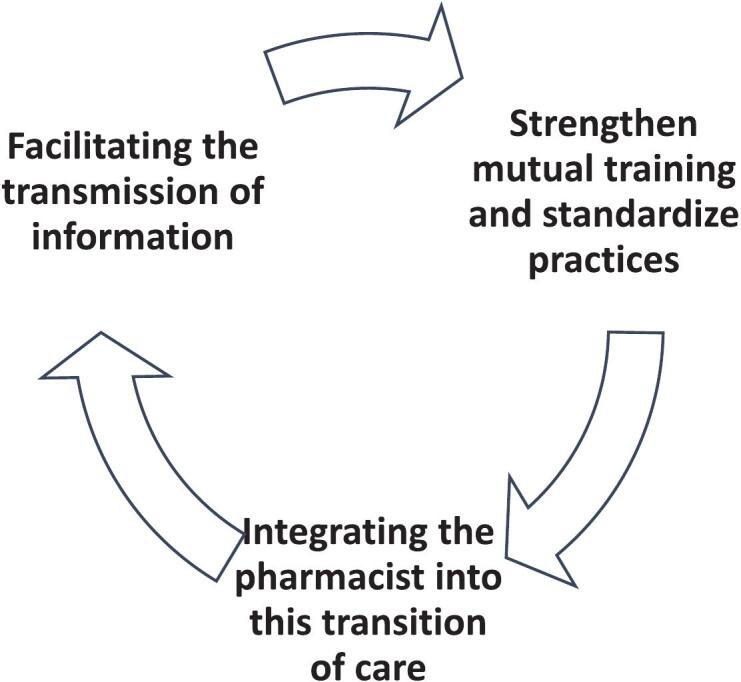
Areas to optimize patients' medication management safety during the transition.

The areas for improvement identified HCPs are synthesized in Fig. 1.

Five onco-hematology professionals expressed that having **both units located on the same hospital site** would be the ideal solution to ensure a safe transition of care.

*“I think it would be easier for the patient because he's at the center of it all. Having to stabilize and transport him, then transfer to a different team… all of that limits his chances.”* HC6.

#### 3.3.1 Facilitating the transmission of information

Improved access **to patient** records and lab results would enable quicker decision-making and reduce the need for duplicate testing.

*“To avoid taking another blood sample when we know the child had a CBC done in the morning—but since we don't have access, it's faster to redo the test.”* HC1.

Three ICU physicians and three ICU nurses, as well as two onco-hematology nurses, mentioned the idea of having **more visual and synthetic discharge** reports, with an hourly schedule and a history of treatments already administered and those to come.

*“I'd like a tool that extracts a much more visual, accessible sheet—not ten pages long—showing prescribed treatments, administration times, and when the last dose was given. That would be ideal.”* HC1.

A nurse from each unit proposed developing a **shared nurse handoff report** tailored to their frequent collaboration and separating nursing handoff documents from medical transmissions, making them easier to locate and act upon patient arrival.

*“Maybe separate medical and nursing handoffs, so we're immediately aware of the child's overall care, including medications.”* HC4.

Six onco-hematology professionals emphasized the need for **more practical information** on drug administration:

*“Sometimes we're missing the exact injection times for medications that can't be mixed. We need basic info—when and where it was given, the route of administration, and the timing”.* HC6.

Additionally, two ICU nurses and one onco-hematology nurse emphasized **that understanding the specific conditions** under which children accept or refuse medications could help tailor care strategies. As one nurse explained*“Some children have difficulties or negotiate a lot before taking medications. It's important to know the strategies that work for each child”* HC5.

Two ICU and four onco-hematology professionals suggested implementing a **verbal transition meeting**. This would help convey nuanced information, “silly little things” (HC6), that may not be captured in written reports and could save time during care delivery.

*“I need a phone call with someone who can brief me on the patient just before discharge—tell me what happened that morning, what meds were given, any special instructions. A real handoff”* HC16.

Four onco-hematology professionals highlighted the importance of receiving information on the **psychosocial aspects of the ICU stay**, such as parental distress or child behavior.

*“Things that aren't medical—like if the parent was emotionally distressed or the child had behavioral issues—those aren't usually documented but are important to know.”* HC18.

#### 3.3.2 Strengthening training and standardizing practices

An ICU nurse suggested **visiting the onco-hematology unit** to better understand its workflow. Another recommended participating in **institutional training programs**. Four onco-hematology professionals advocated for formal and informal joint training initiatives between the two teams.

*“The ICU team should attend refresher training throughout the year to understand what we expect and deliver better care. And they should come observe our practices here to understand our specific needs.”* HC20.

One pharmacist noted confusion between the chemotherapy preparation unit and the central pharmacy, which sometimes complicates pickup and delivery logistics. They recommended either simplifying the process or **providing targeted training** to ICU staff.

Two pharmacists emphasized the need to **better understand onco-hematology protocols**—not only for chemotherapy but also for routine management outside of cancer treatment.

*“We need access to protocols and guidance to validate orders. I'm not sure we fully understand all of their clinical practices.”* HC11.

Four professionals expressed that ICU staff should be trained in these protocols.

Three ICU professionals and one onco-hematology professional highlighted the value of **standardizing certain procedures**—such as mouthwash regimens and IV drug compatibility.

*“We need to have common procedures. To reassure parents and also to have the same practices.”* HC17.

#### 3.3.3 Integrating the pharmacist into the transition of care

Three pharmacists interviewed stressed the need to **be included in communication** during transitions. This would allow them to anticipate chemotherapy preparations and manage requests for special treatments. One proposed creating a “standard patient file” or “template email” (HC11) for ICU transfers.

“*Just call a little earlier—give us a heads-up if a patient is being transferred. Sometimes we're managing weekend chemo preparations at the last minute, which could be avoided with better planning”* HC13.

Two ICU professionals and two pharmacists highlighted the importance of **knowing which medications are required in advance**, especially for drugs that are difficult to obtain on short notice.

*“Getting that info just before transfer helps us prepare. We need to know what the nurses should get ready—ventilatory support, special meds to order”* HC9.

Two ICU physicians and one pharmacist recommended that onco-hematology **prescriptions be reviewed by a pharmacist** after ICU admission. Given the complexity and volume of medications, this would enhance safety.

Three onco-hematology professionals described their **pharmacist as a point of contact**, particularly for chemotherapy coordination.

*“Our specialist (onco-hematology) pharmacist sends the prescription to his pharmacist colleague at the (other facility). For the chemotherapy drug, so it can be prepared”* HC23.

Two ICU professionals noted the need for an “**onco-hematology pharmacist liaison**” (HCP1) to assist with questions about pediatric formulations or drug interactions.

*“We need someone from pharmacy who can work closely with us—observe our workflows, understand our dilutions, identify challenges, and answer our questions. That would be invaluable.”* HC2.

One pharmacist recommended **implementing medication reconciliation** for patients transitioning between units, given the high risk of error and the complexity of care.

*“For onco-hematology patients, this would be especially valuable. Their care is highly specialized and complex.”.* HC11.

## 4 Discussion

To our knowledge, this is the first study to specifically explore the perceptions, experiences, and practices of healthcare professionals regarding medication management during transitions from conventional hospitalization to the ICU in pediatric onco-hematology and in the other way.

According to the participants in our study, ICU admission represents one of the most critical and complex transition points, particularly in emergency contexts. In contrast, ICU admissions for ATMP administration or planned discharges were perceived as less challenging, largely due to greater patient stability and the absence of emergency conditions. Emergency situations were associated with additional major organizational barriers.

Our findings identify actionable strategies to improve medication management during this particularly at-risk transition, which are similar than expert consensus about organization of in-hospital management of life-threatening emergencies. Three elements emerge as essentials: information diffusion, healthcare professionals training and standardization, integrating the pharmacist into this transition of care.

Communication challenges encompassed both technological constraints and practical issues related to information transfer. The interconnectivity patient software systems between two sites of the same center is primordial, in order to have complete access to information needed. This is an institution specific barrier which persist because of technical problems. Even if it is a key numeric challenge for this transition in our institution, it can be found for other moments of the care pathway, by instance the hospital discharge care transition. In our study, inadequate communication—particularly regarding nursing information—was reported to negatively affect care quality and to increase the risk of errors and also stress. Nurses expressed a great need for structured support. This can be answered by standardization of the nursing communication, with verbal exchanges to capture patient-specific nuances as support medication adherence and written handover tools. Nurses demand also training about the other specialty which could begin with observation of the other team's workflow. That will help to support practice transition standardization. Similar findings have been reported in the literature, highlighting the association between nurse-to-nurse communication, patient safety, and care quality.^6^, ^7^, ^8^, ^9^, ^10^, ^11^, ^12^ Hutt et al., in a study on transitions between hematopoietic stem cell transplant units and pediatric ICUs, also recommended structured meetings between nurses from both departments to enhance continuity of care, as well as targeted staff training as essential measures to support patient safety.^13^ In the adult literature, the introduction of a liaison nurse has been described as a supportive intervention,^14^ particularly when the role is available 24/7. However, implementing such a model can be challenging in practice, whereas protocolized nursing transfers and trainings appear less costly strategies.

All those improvement areas have the same aim: reducing adverse events. Involving clinical pharmacist in pediatric ICU could answer this aim. Even if implementing such a model could be challenging as well, it could be interesting to improve provider knowledge, offer economic benefits^15^, ^16^, ^17^ and improve care outcomes by medication reconciliation during ICU transfers.^18^ Our findings show that ICU professionals would welcome pharmacist, and even more they asked him. In our study, pharmacists were seen as important allies in optimizing communication between physicians and nurses across both units. Their role could include conducting medication reconciliation, generating clear visual summaries of current therapies, and standardizing protocols for drug preparation and administration across the ICU and onco-hematology teams.^15^, ^16^, ^17^, ^18^ A liaison pharmacist role could address expectations related to medication availability and safety. Although the permanent integration of a clinical pharmacist into the pediatric ICU may be costly, targeted or intermittent interventions appear feasible and essentials.

Although this study focused on medication management, many participants raised broader concerns about the patient and family experience highlighting an issue of particular importance for healthcare providers. ICU transitions were described as stressful for families due to changes in care environment, unfamiliar staff, and a perceived drop in continuity of care—particularly in the context of geographically separated units. Consistent with previous findings, transfer to the ICU was described as a potentially traumatic experience for parents.^18^ Inadequate coordination between professionals is central in the communication with families: the importance is to re-inform and reassure families. Parents are still in ICU integral members of the pediatric care triangle, alongside the child and healthcare professionals. Healthcare professionals need to acknowledge the central role that parents play for their child's care and communicate with families about it. This aligns with other research on parental perspectives during transitions from ICU to general care, which has consistently highlighted the importance of information sharing, active parental involvement, and sensitivity to environmental changes.^19^, ^20^, ^21^, ^22^ Parents should not be involved in their child's care to compensate for shortcomings in the healthcare system, but rather because they have an essential role as care partner. Maintaining this parental role during hospitalization is important for the child's well-being. This allows showing parents that their children are not objects of care but individuals.^12^ Enhancing interprofessional communication between ICU and onco-hematology teams during these traumatic transfers ultimately aims to make the transition less difficult for families. These findings suggest the value of conducting a complementary study to explore parents' perspectives directly, providing a more systemic understanding of transition experiences.

Some of the elements identified, particularly those related to the geographical separation may be specific to this institution and not generalizable to other healthcare settings as the lack of medicalized transport. However, certain points, such as improving communication by trainings and standardizing practices could be transferable to other organizations and also other transitions. Those are common challenges also observed in the study by Hutt et al.,^13^ including parental distress related to poor coordination of care between teams. When compared with other pediatric care transitions, such as hospital discharge, transitions to the ICU in emergency contexts are considerably more complex, as planning and anticipation are often not possible. This likely explains why protocol standardization appears to be particularly valuable in this setting. Also, implementing pharmacist is a key challenge not specific to our institution and to this care transition. However, pediatric ICU and even more transition to ICU involved a challenging medication practices. If the pharmacist is seen as a facilitator of quality care, pediatric ICU needs it. He will facilitate information diffusion, training and practices standardization about proper use of medications.

### 4.1 Methodological limitations

This study has several limitations. First, data analysis was conducted manually, without the use of qualitative analysis software. However, this was a deliberate choice, as the software available primarily supports guided manual coding rather than fully automated analysis.

Second, the study was conducted at a single center, divided into two separated sites. Multicenter research would be valuable to determine whether these issues are consistent across institutions or vary by setting.

In our methodology, we chose to conduct separated data collections and analysis by two separated interviewers. This approach allowed interviews to be carried out during the same period for both groups—ensuring comparable timing and context—. Limited the learning bias of interviewer was the principal aim of this approach. As it, each ecosystem has a proper comprehension. However, a limitation of this approach was the potential risk of each interviewer subjectivity. To minimize this risk, both interviewers were trained simultaneously by the same expert researcher, using a standardized methodology. In addition, an expert researcher supervised the analytical process and reviewed and cross-validated both analyses to ensure consistency and limit subjective interpretation. Separate analysis by group were intentionally conducted rather than a cross-group thematic comparison in order to minimize the influence of one group's perspectives on the other. Finally, discussions with both the ICU and onco-hematology teams were conducted to enhance the credibility and trustworthiness of the interpretations.

## 5 Conclusion

This study explored the perceptions, experiences, and practices of healthcare professionals involved in transitions of care between pediatric onco-hematology and intensive care units, with a specific focus on medication management. Several barriers were identified, including differences in software systems, challenges in documentation and transfer ergonomics, and variations in care protocols between the two units. More broadly, participants highlighted the unique complexity of managing onco-hematology patients and the essential role of parents in pediatric care.

The study also revealed actionable opportunities for improvement: enhancing the transmission of clinical and psychosocial information, strengthening cross-training between teams, standardizing care practices, and involving clinical pharmacists more actively in the transition process. These changes could help improve patient safety, streamline workflows, and reduce stress for both families and healthcare providers.

## CRediT authorship contribution statement

**C. Baudy:** Writing – review & editing, Methodology, Investigation, Formal analysis, Data curation. **O. Kouassi:** Writing – review & editing, Formal analysis, Data curation. **M. Philippe:** Writing – review & editing, Investigation. **C. Gervaise:** Supervision, Project administration, Investigation. **S. Remy:** Writing – review & editing, Validation, Resources, Investigation. **E. Javouhey:** Writing – review & editing, Validation, Investigation. **B. Dumont:** Writing – review & editing, Validation, Investigation. **C. Halfon-Domenech:** Writing – review & editing, Validation, Investigation, Conceptualization. **V. Bréant:** Writing – review & editing, Investigation. **D. Hoegy:** Writing – original draft, Validation, Supervision, Methodology, Investigation, Formal analysis, Conceptualization.

## Funding

This study did not receive any specific funding.

## Declaration of competing interest

The authors declare that they have no known competing financial interests or personal relationships that could have appeared to influence the work reported in this paper.
